# Supplementary material for: Deletion of wfs1 Impairs Oligodendrocyte Precursor Cells Dorsal Distribution and Myelination Through the wfs1-hmgcs1 Axis in Zebrafish
Source: Biology (Basel). 2026 Aug 16;15(16):1402. doi: 10.3390/biology15161402 (PMC13510151; doi:10.3390/biology15161402)
Supplement: Supplementary file 1 [file biology-15-01402-s001.zip › File S1-Transcriptome sequencing data and differentially expressed genes (DEGs) in wfs1-- zebrafish at 5 dpf/2.KEGG/wfs1KOvsWT/GSEA/WT/ARGININE_BIOSYNTHESIS/ARGININE_BIOSYNTHESIS(DRE00220).html]

Details for gene set ARGININE\_BIOSYNTHESIS(DRE00220)[GSEA]

|  || Dataset | deg.sample |
| Phenotype | sample.cls |
| Upregulated in class | WT |
| GeneSet | ARGININE\_BIOSYNTHESIS(DRE00220) |
| Enrichment Score (ES) | -0.23561294 |
| Normalized Enrichment Score (NES) | -0.7800743 |
| Nominal p-value | 0.7014028 |
| FDR q-value | 0.8676128 |
| FWER p-Value | 1.0 |
Table: GSEA Results Summary

  

Fig 1: Enrichment plot: ARGININE\_BIOSYNTHESIS(DRE00220)      
 Profile of the Running ES Score & Positions of GeneSet Members on the Rank Ordered List

  

| PROBE | DESCRIPTION (from dataset) | GENE SYMBOL | GENE\_TITLE | RANK IN GENE LIST | RANK METRIC SCORE | RUNNING ES | CORE ENRICHMENT || 1 | 406688\_1 | got2a |  |  | 2167 | 1.300 | 0.0128 | No |
| 2 | 100006303 | gls2a |  |  | 2628 | 1.135 | 0.0689 | No |
| 3 | 101886255 | LOC101886255 |  |  | 2978 | 1.042 | 0.1228 | No |
| 4 | 322614 | arg2 |  |  | 5009 | 0.680 | 0.1014 | No |
| 5 | 100001620 | LOC100001620 |  |  | 6052 | 0.582 | 0.1048 | No |
| 6 | 799963 | gpt2 |  |  | 6058 | 0.581 | 0.1408 | No |
| 7 | 317737\_1 | glud1a |  |  | 6910 | 0.499 | 0.1451 | No |
| 8 | 406330 | got1 |  |  | 8454 | 0.406 | 0.1219 | No |
| 9 | 336473\_1 | glulb |  |  | 9542 | 0.330 | 0.1083 | No |
| 10 | 403086 | gpt2l |  |  | 11238 | 0.231 | 0.0695 | No |
| 11 | 794667 | otc |  |  | 11618 | 0.210 | 0.0706 | No |
| 12 | 564147 | glsa |  |  | 12468 | 0.166 | 0.0543 | No |
| 13 | 791730 | got1l1 |  |  | 13108 | 0.135 | 0.0426 | No |
| 14 | 406330\_1 | got1 |  |  | 14060 | 0.089 | 0.0183 | No |
| 15 | 563654 | nos2b |  |  | 15462 | 0.024 | -0.0242 | No |
| 16 | 797748 | glsl |  |  | 15795 | 0.007 | -0.0342 | No |
| 17 | 393970 | acy1 |  |  | 16923 | -0.032 | -0.0676 | No |
| 18 | 570368\_1 | arg1 |  |  | 17347 | -0.048 | -0.0779 | No |
| 19 | 447864 | ass1 |  |  | 17996 | -0.075 | -0.0935 | No |
| 20 | 335974 | got2b |  |  | 18318 | -0.090 | -0.0980 | No |
| 21 | 100000775 | glula |  |  | 19256 | -0.133 | -0.1191 | No |
| 22 | 373092 | glud1b |  |  | 20882 | -0.209 | -0.1572 | No |
| 23 | 556445 | gls2b |  |  | 21211 | -0.226 | -0.1534 | No |
| 24 | 566165 | glulc |  |  | 21829 | -0.258 | -0.1568 | No |
| 25 | 564746 | glsb |  |  | 23111 | -0.335 | -0.1762 | No |
| 26 | 317737 | glud1a |  |  | 25006 | -0.446 | -0.2079 | Yes |
| 27 | 393423 | asl |  |  | 25054 | -0.451 | -0.1813 | Yes |
| 28 | 557716 | nags |  |  | 25514 | -0.486 | -0.1655 | Yes |
| 29 | 60658 | nos1 |  |  | 26051 | -0.533 | -0.1492 | Yes |
| 30 | 557716\_1 | nags |  |  | 26881 | -0.617 | -0.1368 | Yes |
| 31 | 100148522 | si:ch211-217a12.1 |  |  | 27762 | -0.711 | -0.1203 | Yes |
| 32 | 100006303\_1 | gls2a |  |  | 27798 | -0.715 | -0.0769 | Yes |
| 33 | 404036 | nos2a |  |  | 28157 | -0.765 | -0.0405 | Yes |
| 34 | 555623 | cps1 |  |  | 28996 | -0.919 | -0.0097 | Yes |
| 35 | 406688 | got2a |  |  | 30634 | -1.617 | 0.0394 | Yes |
Table: GSEA details [plain text format]

  

Fig 2: ARGININE\_BIOSYNTHESIS(DRE00220)      
 Blue-Pink O' Gram in the Space of the Analyzed GeneSet

  

Fig 3: ARGININE\_BIOSYNTHESIS(DRE00220): Random ES distribution      
 Gene set null distribution of ES for **ARGININE\_BIOSYNTHESIS(DRE00220)**
